# A low-cost perfusion heating system for slice electrophysiology

**DOI:** 10.1038/s41598-024-79856-4

**Published:** 2024-11-18

**Authors:** Matthijs Dorst, Koen Vervaeke

**Affiliations:** 1 Institute of Basic Medical Sciences, Section of Physiology, University of Oslo, Oslo, Norway; 2 Department of Neurscience, Karolinska Institutet, Solna, Sweden

**Keywords:** Biological techniques, Patch clamp, Cellular neuroscience, Ion channels in the nervous system

## Abstract

Temperature-critical applications, such as patch-clamp electrophysiology, require constant perfusion at a fixed temperature. However, maintaining perfusate at a specific temperature throughout various applications requires heaters or coolers with integrated feedback systems, which has historically increased complexity and cost. This makes such systems prohibitively expensive in research environments with lower funding rates, particularly in developing countries. We developed a custom temperature control system that relies on off-the-shelf components and few custom parts, which can be easily produced with common tools. Our system can be built for less than $30 and maintains a set perfusate temperature within 0.4 °C while introducing negligible electrical interference. Using this system, we demonstrate that Striatal Medium Spiny Neurons exhibit increased membrane resistance, longer membrane time constants, lower firing rates, and increased rheobase current at room temperature compared to physiological temperature.

**Supplementary Information:**

The online version contains supplementary material available at 10.1038/s41598-024-79856-4.

## Introduction

Neurons are exquisitely sensitive to temperature^1–4^, and the body goes to great lengths to maintain thermal homeostasis. Therefore, to mimic physiological conditions, in vitro experiments are typically performed at approximate body temperatures between 33 °C and 35 °C.

Maintaining this temperature during patch-clamp recordings is complicated by the need to continuously perfuse freshly oxygenated artificial cerebrospinal fluid (ACSF) at the desired recording temperature^5^. It is relatively straightforward to heat up ACSF in a water bath or on a heating pad; however, since ACSF must be continuously perfused over the slice or cell culture, typically through thin tubes, this can introduce variability when the ACSF is transported to the recording chamber. Furthermore, large changes in temperature can affect temperature-sensitive components in the ACSF, and influences the solubility of oxygen^6^. Therefore, modern temperature control systems continuously monitor the temperature in the recording chamber and apply heating to the ACSF in or just before reaching the recording chamber. Such in-line heating solutions have become the standard in high-quality electrophysiological research, so manufacturers now offer holistic solutions whereby the heating system is integrated within the microscope. However, two issues exist with these solutions: they are often difficult to repair and service by the end-user, and they can be prohibitively expensive, costing up to several thousand dollars for a complete heating system optimized for life-science applications. This latter aspect significantly reduces their utilization in environments where the trade-off between recording quality and cost favors affordability. While custom heating and cooling systems have been designed for patch-clamp electrophysiology, these are typically employed in scenarios where more complex or fine-grained control necessitates a more advanced solution than what is commercially available^7–9^.

High costs have long been a critical damper on the accessibility of science^10^. Our solution to this inaccessibility of high-quality temperature control systems follows in the footsteps of other low-cost, user-buildable scientific equipment. Scientific equipment has been developed to lower costs through 3D printing^11,12^, utilizing commercially available components, and even through the use of LEGO^®^^13^. In this vein, we developed a custom inline perfusate heating solution that uses off-the-shelf components, 3D printing, and one easily machinable part. Our system can be built for less than $30 in parts and materials and requires minimal access to tooling or electrical engineering skills. It utilizes a commonly available Peltier element to heat or cool the perfusate. This Peltier element introduces no significant electrical interference^9^, making this solution ideal for electrophysiology research. Our system can maintain recording temperatures within a maximum deviation of 0.4 °C of the desired temperature over prolonged durations, with a standard deviation of just 0.2 °C. We used this system to perform reproducible, high-quality patch-clamp experiments.

The effects of temperature on neuronal activity are complex; while an extensive body of research exists on the impact of temperature on individual ion channels^14^and TRP-channels in particular^15^, far less data is available on how these effects interact at the neuronal level^16,17^. By performing ex vivo patch-clamp recordings of Medium Spiny Neurons (MSNs) in the mouse Striatum, we demonstrate here for the first time how recording temperature affects the membrane properties of Striatal MSNs.

## Results

### Component selection and validation

**Fig. 1:**
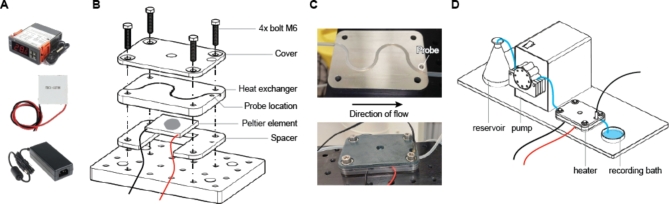
Design and assembly of the perfusion heating system.** (A)** From top to bottom: the electrical components of the heater are comprised of a temperature controller, Peltier element, and power adapter. **(B)** Heating system assembly. **(C)** Top: the heat exchanger with perfusion tubing installed in the cut groove. Note the probe location is in close proximity to the exit point of the perfusion tube. Bottom: the fully assembled system is mounted on an aluminium breadboard. **(D)** A complete perfusion system for patch clamp electrophysiology requires a reservoir, pump, and heater.

We build a temperature regulator using two readily available components: an STC-1000 temperature controller and a TEC1-12706 Peltier element (Fig. 1A). The temperature controller can be configured to maintain a specific temperature within a configurable range and opens or closes a heating or cooling relay as required. We opted for a 12 V, DC version of both the temperature controller and Peltier element, as 12 V power supplies are safe and easily available. Indeed, the temperature controller can also be powered by a 12 V battery if desired for ultra-low noise applications.

Using a Peltier element for heating reduces power requirements, and thus less potential electrical noise compared to a thermoresistive heating element of comparable output. Furthermore, a Peltier element can be mounted in the inverse orientation to cool the perfusate instead of heating it, increasing the flexibility of this design. The TEC1-12706 Peltier element is rated for a maximum temperature difference of 67 °C and draws a maximum of 6.0 Amperes, which fits well within the operational parameters of patch-clamp electrophysiology. The Peltier element may be readily swapped out for one with a higher capacity for applications requiring higher flow rates, or larger temperature differences.

### Assembly of Peltier element and heat exchanger

Care must be taken when mounting the Peltier element, as this component is brittle and may easily fracture. Therefore, we designed a spacer / mounting bracket to reduce mechanical stress on the Peltier element^18^ and evenly distribute mounting pressure (Fig. 1B, C). When 3D printing this component, the infill, or amount of internal structure, should be reduced to a minimum to maximize thermal insulation between the hot and cold sides of the Peltier element.

To reduce thermal losses, we designed and printed a cover plate using the same process. Extra spacing is provided for the thermal probe, which informs the STC-1000 temperature controller of the heat exchanger’s temperature near the exit point of the perfusate.

The heat exchanger represents the only part that requires machining. This part should be constructed using a material with high thermal conductivity for optimal results, e.g. aluminium. When machining this part from raw stock, holes may be drilled oversized to account for dimensional inaccuracies, as the exact mounting position is unimportant. Alternatively, the cover plate can be used as a guide to ensure correct hole locations (Fig. 1C, bottom).

A groove should be cut into one face of the heat exchanger to hold the perfusate flow tube. To optimize heat transfer, thin-walled tubing is preferred, taking care to select a material with low oxygen permeability^19^. The groove should be cut slightly narrower than the outside diameter of the perfusion tubing, but at least 0.5 mm deeper than the tubing diameter for optimal contact. For a perfusion tube of 3 mm outer diameter, we used a 3 mm wide groove cut to a depth of 3.5 mm, as illustrated in Fig. 1B, C and Supplementary Fig. 1. While the exact path is not critical, the illustrated pattern represents an acceptable trade-off between maximizing path length while avoiding sharp bends, which could slow down or stall the perfusate. Finally, a 4 mm hole should be drilled to hold the temperature probe included with the STC-1000 temperature controller (Fig. 1B-D).

Parts should be mounted as illustrated in Fig. 1B. To increase heat transfer, a thermally conductive material, e.g., a generic thermal paste, should be applied to both sides of the Peltier element. Ensure the Peltier element is oriented correctly for the desired application; typically, heat is transferred toward the unmarked side. After the perfusion tube is pressed into its receptive groove, the system can be screwed down onto an optical breadboard or air-table. Care must be taken to gradually tighten each bolt, ensuring no excessive force on any side of the system.

Following successful mounting, a 12 V power supply of at least 2 Amp capacity can be connected to the Peltier element. The ground lead may be connected directly to the Peltier element, while the positive lead should be interrupted by the relay marked for “heating” or “cooling” on the STC-1000, depending on the desired application (Supplementary Fig. 2). For the TEC1-12706, the maximum driving current should not exceed 6.0 Amps. A separate 12 V power supply should be connected to the STC-1000, and the heat controller may then be configured according to its manual.

**Table 1:** Cost of components

Item	Supplier	Cost (in USD)
STC-1000 temperature controller	AliExpress, China	4.57
TEC1-12706 thermocouple	AliExpress, China	1.84
12 V 3 A power supply	AliExpress, China	4.21
Thermal paste	AliExpress, China	4.47
Cover (3D printing filament cost)^18^	Prusa Research, Czechia	0.69
Spacer (3D printing filament cost)^18^	Prusa Research, Czechia	0.95
Aluminium stock^18^	local supplier, Norway	~ 10.00
**Total cost**		**26.73**

### System cost

An overview of parts with an estimated cost at time of writing is provided in Table 1. If aluminium stock is available, the total cost in materials comes to approximately $27. Since the parts used are widely available, cheaper local alternatives may exist and unit cost will vary accordingly.

Note that machining and 3D printing costs are not included in this cost estimate. Commercial 3D printing services exist; the cost to print the spacer and cover with Shapeways (Shapeways, Inc., USA) is currently $18.68 and $25.41, respectively. Likewise, a commercial CNC machining service may be used to produce the heat exchanger. With Hubs (Hubs B.V., The Netherlands), the cost of producing the heat exchanger in aluminium is currently estimated at $100.32.

### Thermal stability

**Fig. 2:**
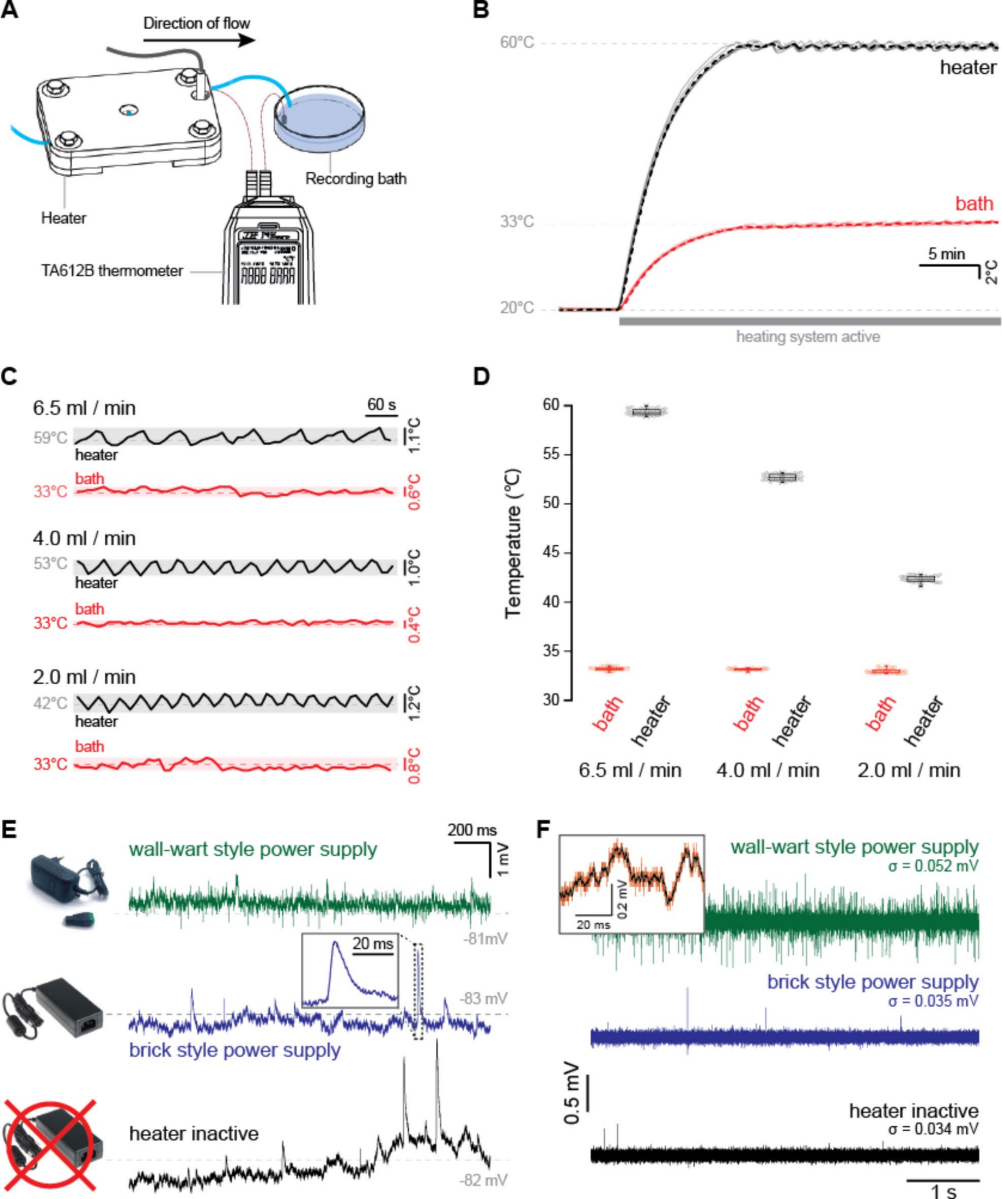
Assessment of thermal stability and electrical interference.** (A)** Schematic illustration of the recording setup. A calibrated multi-channel thermometer was used to map the temperature over time as the system is activated. **(B)** Repeated heating cycles from room temperature to physiological temperatures at 6.5 ml/min demonstrate consistent heating performance. Solid lines: average (*n* = 6 repetitions), dashed lines: individual recordings. **(C)** Example bath and heater temperature recordings in equilibrated state for different flow rates. Scale-bars indicate recorded temperature range during a 10-minute recording. **(D)** Overview of the recordings in C. Box plots denote interquartile and maximum range. **(E)** Example electrical noise profile with excitatory post-synaptic potentials (EPSPs). Inset: higher magnification of an example EPSP marked by the dotted box. **(F)** isolated noise during patch-clamp recordings with a wall-wart style power supply, a brick style power supply, and with the heating system disconnected. Inset: the unfiltered (red) and filtered (black) signals were subtracted to isolate high-frequency noise in the recording.

To assess whether our heating solution could provide a sufficient level of thermal stability, we recorded the temperature at the heat exchanger and the temperature in the recording bath at 10-second intervals using a TA612B multichannel thermometer (TASi group, United Kingdom). The heating system was calibrated for a bath temperature of 33 °C at a high (6.5 ml / min), medium (4 ml / min), and low (2 ml / min) flow rate (Fig. 2A).

After recording a baseline at room temperature, the heater was activated and reached operating temperature after approximately ten minutes for the high flow-rate scenario of 6.5 ml / min. The system was left to equilibrate for another ten minutes before thermal stability was assessed. During the subsequent 5-minute recording period, bath temperature was maintained at an average of 33.1 ± 0.2 °C (*n* = 6 repeats, mean ± std), within the range of [32.7, 33.5] °C, as shown in Fig. 2B. Minor changes in initial temperature of the perfusate did not affect thermal stability, nor the offset between the temperature at the heat exchanger versus temperatures in the recording bath.

At the high flow-rate scenario, the heating block eventually settled at an average temperature of 59.6 ± 0.4 °C (mean ± std), within the range of [58.9, 60.4] °C (*n* = 6 repeats).

Changing the flow rate of the perfusate altered the difference between bath temperature and heater temperature, as shown in Fig. 2C, D. At higher flow rates, the heater should be set at a higher target temperature to maintain physiological temperatures in the recording bath. Therefore, a look-up table should be created to map this correlation between bath temperature and flow rate. The increased temperature requirement for higher flow rates also increases the ratio of time spend heating up (supplementary Fig. 3) and therefore cycle time, though this does not affect thermal stability (Fig. 2C, D).

### Electrical interference

To test electrical interference, we calculated a baseline for electrical noise in the system during whole-cell patch-clamp recordings with the heating unit briefly disabled, and compared this to a scenario where the heating unit was turned on and actively heating. Example traces are shown in Fig. 2E. Noise levels were calculated from a current-clamp recording of a patched MSN at rest, at a stable holding potential but receiving random excitatory post-synaptic potentials (EPSPs). Briefly, the recorded trace was put through a 1-D digital filter utilizing a rational transfer function, whereupon noise was calculated as the difference between the raw and filtered trace (Fig. 2F, inset). This produced an estimate of the high-frequency noise present in the recording, as illustrated in Fig. 2F.

Initial testing produced high-frequency artifacts during the heater-on scenario. We determined that the wall-wart style power adapter, used to drive the Peltier element, was responsible for producing this noise, and replaced this with a brick-style power adapter of similar capacity. With this brick-style power supply, noise levels decreased to the point where we could no longer detect a difference between the heating on versus heating disconnected scenarios. This is likely due to the more robust build quality of the larger brick-style power adapter, and greater physical distance between its input and output sides. These results suggest that the selection of a low-noise power supply is critical for achieving a low electrical noise floor.

Given this exceptional performance in terms of heating stability and virtually undetectable levels of electrical interference, we conclude that this heating unit is well suited for patch-clamp electrophysiology, and other applications that require stable temperatures and low electrical noise.

### Thermal modulation of Striatal Medium Spiny Neurons alters their neurophysiological properties

**Fig. 3:**
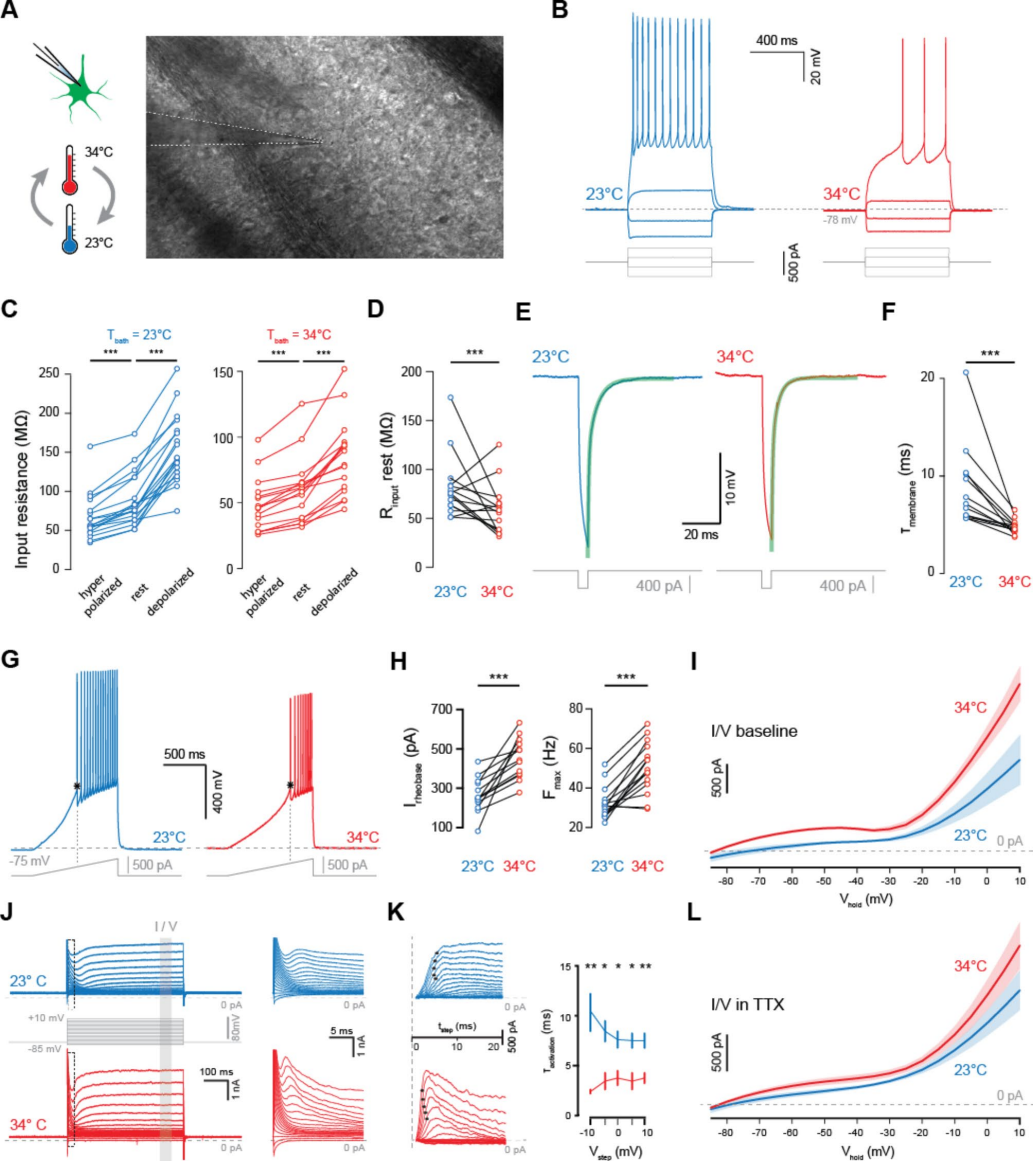
Membrane properties of Striatal Medium Spiny Neurons are temperature dependent.** (A)** Whole-cell patch clamp recordings were made from Striatal MSNs at 23 °C and 34 °C. Right: wide-field high-contrast microscopy image of the patch clamp recording pipette and target MSN. **(B)** Identical step current injections highlight the temperature dependence of MSN membrane properties. **(C)** Input resistance increases with depolarizing membrane potential, both at 23 °C and 34 °C. **(D)** At rest, input resistance is significantly higher at 23 °C compared to 34 °C (*p* = 0.00064, *n* = 15 MSNs, paired-samples T-test). **(E)** The membrane time constant was assessed by fitting a double exponential curve to the repolarization phase following a 5 ms hyperpolarizing current pulse. **(F)** Membrane time constant is significantly slower at 23 °C (*p* = 0.0012, *n* = 14 MSNs, paired-samples T-test). **(G)** Action potential onset and Maximum firing rate were determined by injecting a linear depolarizing ramp current. **(H)** Rheobase current and maximum firing rate were significantly lower at 23 °C (*p* = 0.00019, *n* = 13 MSNs, and *p* = 0.000011, *n* = 14 MSNs respectively, paired samples T-test). **(I)** Steady-state current-voltage relationship at 23 °C and 34 °C between − 85mV and + 10mV assessed by voltage clamp recording. Shaded areas represent SEM. **(J)** Left: Example voltage-clamp recording in the presence of TTX. Grey bar marks the 50ms domain where I/V relationship was determined. Right: Higher magnification of the area indicated by the dotted box. **K.** Left: The remaining currents in (**J**) following subtraction of estimated leak-current. Asterisks indicate the time constant of the remaining current, as the point where I = 1–1/e of I_max_. Right: the voltage-sensitive currents exhibit significantly slower time constants at 23 °C compared to 34 °C, at −10mV to + 10mV (*p* = 0.003, 0.034, 0.034, 0.011, 0.007, independent-samples Mann-Whitney U test). **K.** As **I**, in the presence of TTX.

We performed in vitro whole-cell patch-clamp recordings of Striatal MSNs (Fig. 3A, B). Membrane properties obtained through current-clamp recordings performed at room temperature and physiological temperatures were compared against reported properties of these neurons^20–23^, including input resistance (Fig. 3C, D), membrane time constant (Fig. 3E, F), rheobase current, the minimal current required to trigger an action potential, and maximum firing rate (Fig. 3G, H). Additionally, we performed voltage-clamp recordings under baseline conditions and in the presence of 1 µM tetrodotoxin (TTX)^24^ to investigate the thermal modulation of voltage-gated sodium- and potassium channels (Fig. 3I-L).

Input resistance was determined by applying a hyperpolarizing or depolarizing current pulse while maintaining the membrane potential near − 75 mV (Fig. 3B). The resistance increased with increasing membrane potential, and was significantly lower at 34 °C compared to 23 °C over the tested range of membrane potentials: R_hyperpolarized_ was 67.0 ± 7.8 MΩ at 23 °C versus 44.9 ± 4.1 MΩ at 34 °C (*p* = 0.010), R_rest_ was 88.0 ± 8.6 MΩ at 23 °C versus 55.0 ± 4.7 MΩ at 34 °C (*p* = 0.00064), and R_depolarized_ was 152.9 ± 12.3 MΩ at 23 °C versus 81.2 ± 7.0 MΩ at 34 °C (*p* = 0.000039, for all tests *n* = 15 MSNs, mean ± SEM, paired samples T-test) (Fig. 3C, D).

The membrane time constant was determined by applying a brief (5 ms) hyperpolarizing current pulse from rest and fitting a double exponential to the relaxation phase^25^, in order to model the fast and slow components of the membrane capacitance, focusing here on the slow component (Fig. 3E). Membrane time constants were significantly slower at room temperature at 8.76 ± 1.07 ms, compared to 4.78 ± 0.73 ms at physiological temperatures (*p* = 0.0012, *n* = 14, mean ± SEM, paired samples t-test) (Fig. 3E, F).

A ramp current was applied to determine the minimal current necessary to induce an action potential. The inflection point of the first action potential was used as the onset time (Fig. 3G), and the inter-spike interval was used to determine the maximum firing rate during a ramp current. Commensurate with increased input resistance, rheobase current was lower at 264 ± 25 pA during room temperature recordings compared to 452 ± 29 pA at physiological temperatures (Fig. 3G, H) (*p* = 0.00019, *n* = 13 MSNs, mean ± SEM, paired samples T-test). Likewise, as predicted by the longer membrane time constant, the maximum firing rate at room temperature was significantly lower at 32.2 ± 2.1 Hz, compared to physiological temperature, 50.4 ± 3.3 Hz (*p* = 0.000012, *n* = 15 MSNs, mean ± SEM, paired samples T-test) (Fig. 3G, H).

Voltage clamp recordings were used to assess the temperature sensitivity of voltage-gated currents. MSNs were held at −75mV, then stepped to −85mV to + 10mV in 5mV steps for 500ms (Fig. 3J). The mean current at 400ms to 450ms into the voltage step was used to determine the steady-state I/V relationship (Fig. 3I, J). At baseline, the I/V relationship shifted substantially from 23 °C to 34 °C. Next, tetrodotoxin was bath applied at 1 μm^24^ to remove voltage gated sodium currents. Leak currents were modelled based on the − 70mV to −60mV voltage steps and subtracted from the I/V response to estimate K_V_ currents (Fig. 3K, left). The time constant of this current was determined by finding the spot where I_KV_ reached 1-(1/e) of I_max_, indicated by asterisks (Fig. 3K, left). This demonstrates a significantly slower K_V_ time constant at 23 °C, for membrane potentials between − 10mV to + 10mV (Fig. 3K, right). As before, the steady-state I/V relationship in voltage-clamp mode was shifted up from 23 °C to 34 °C in the presence of TTX (Fig. 3L), demonstrating the temperature sensitivity of both Na_V_ and K_V_ currents in Striatal MSNs.

## Discussion

Affordability is closely related to the accessibility of science. In recent years, the scientific community has designed many low-cost alternatives to expensive scientific equipment. In this vein, we sought to develop a low-cost heating solution to perform patch-clamp electrophysiology. By selecting low-cost, readily available components, we ensured our heating solution is within the financial reach of most research labs. Furthermore, to avoid relying on expensive custom-made components, we designed our heating solution to use 3D printed parts. While our solution does require one machined part, this part can be readily produced with simple tools and does not necessitate a high degree of accuracy, which typically drives up the cost and skill required to produce such parts. Thus, we are confident our solution can be easily replicated by other research groups.

A major requirement for a custom perfusion heating device is that it must outperform more straightforward solutions, such as using a heating plate to warm up the ACSF prior to circulation. Compared to heating the perfusate in a water bath or on a heating plate, our solution provides a more stable recording temperature, is less susceptible to fluctuations in room temperature, requires considerably less power, and crucially, does not require pre-heating the perfusate to excessively high temperatures. Heating the perfusate closer to the recording chamber carries the risk of inducing errand electrical noise; we avoid this by using a low-voltage thermocouple, which is further shielded by the large aluminium heat exchanger. In practice, electrical noise from our system is sufficiently low for all but the most sensitive applications of patch clamp electrophysiology.

Compared to commercial solutions, our approach has certain disadvantages: when the temperature probe is positioned on the heat exchanger, bath temperature is affected by the flow rate of the perfusate (e.g. see Fig. 2C, D). Moreover, this temperature differential is non-linearly affected by perfusion rate, necessitating a careful mapping between perfusion rates and heating settings. Due to the high degree of stability and reproducibility of these mappings, the added uncertainty by this temperature differential is minimized, but in scenarios where perfusion rates are dynamically adjusted, this may become problematic. Placing the temperature sensor in the bath will alleviate the issue of this variable temperature differential; however, due to the lag between the heater being activated or deactivated and the perfusate heating up or cooling down, thermal stability in the bath will decrease in this scenario.

A more robust solution would replace the temperature controller by a custom solution that incorporates two probes, one at the heater and one in the bath, to maintain a stable heater temperature while adjusting the target temperature for this heater depending on the reading in the bath. While building such a solution is feasible, it would require custom code and additional custom parts (e.g. an Arduino, temperature sensor module, relay module). This in turn would drastically increase the cost and complexity of the system. In practice, when maintaining a fixed perfusion flowrate, we find this issue to be irrelevant in our applications.

As it takes approximately 10 min to reach physiological temperature, this solution is not immediately suitable for probing fast temperature sensitive channels^26,27^. This could be mitigated somewhat by utilizing a smaller heatsink and higher-capacity Peltier element, but for such experiments, the use of more expensive, purpose-designed heaters is well justified. Likewise, while we could not detect electrical interference from our heater in regular whole-cell patch-clamp recordings, more specialized research investigating significantly smaller currents may not find the noise-floor of our solution adequate.

Finally, we demonstrate that recording electrophysiological parameters should be performed at physiological temperatures to enable comparison to published research. We report dramatic changes in input resistance, time constant, rheobase current, and maximum firing rates between room temperature and physiological temperature. Reported values for MSN membrane resistance range from 53.1 MΩ for D1 MSNs^21^at room temperature following a hyperpolarizing current step, to 151.37 MΩ for D2 MSNs at 34°−35°C at a depolarized membrane potential^20^. Common values for MSN membrane resistance at rest are reported as 128 MΩ at 34°−35°C^23^, 112.1 MΩ at 33 °C^22^(in rats), 85.26 MΩ at 34°−35°C^20^and 124.40 MΩ at room temperature^21^. While this range overlaps with values reported here (47.3 to 88.6 MΩ at 34 °C, 59.9 to 142.6 MΩ at 23 °C), the discrepancy between our results and between and within other studies suggests a critical dependence of input resistance on the exact holding and deviation current, which complicates making an exact comparison.

Greater agreement is found on membrane time constant, reported at 4.84 ms for D1 MSNs to 5.87 ms for D2 MSNs^20^, 7.2 ms for all MSNs^23^, or 2.9 ms for D1 MSNs and 2.3 ms for D2 MSNs^21^. Our result of 4.85 ms falls well within this range, though it is again clear that the literature disagrees on the relative time constant of D1 versus D2 MSNs, and it is peculiar that the smallest reported time constant was recorded at room temperature. This confirms that the electrophysiological parameters of Striatal MSNs, and indeed likely many other neurons, critically depend on a range of recording conditions. Recording temperature significantly influences all parameters tested, illustrating that maintaining a stable recording temperature is critical for comparing results between, and even within studies. Our heating system provides sufficient stability and accuracy to remove at least this factor of uncertainty, yet our results highlight the ongoing challenge in ensuring reproducibility of electrophysiology data.

We conclude that custom building a heating solution dramatically lowers its cost, without sacrificing the ability to perform reliable research. This study provides a unique dataset on how temperature affects the electrophysiological properties of Striatal MSNs, and highlights the importance of careful control over recording conditions.

## Methods

### Patch-clamp electrophysiology

***Slice preparation***: Whole-cell patch clamp recordings were performed as previously described^23^. Briefly, wild-type C57BL/6J mice were anesthetized with isoflurane, before the brain was dissected out and cut into parasagittal slices of 250 μm thickness using a VT1200 Vibrating blade microtome (Leica Biosystems, United States), while submerged in ice-cold cutting solution containing (in millimolar): KCl 2.5, NaH_2_PO_4_ · H_2_O 1.25, CaCl_2_ · 2 H_2_O 0.5, MgCl_2_ · 6 H_2_O 7.5, Glucose 10, NaHCO_3_ 25, and Sucrose 205. Slices were subsequently left to recover for 30 min at 35 °C in artificial cerebrospinal fluid (ACSF) containing (in millimolar): NaCl 125, KCl 2.5, MgCl_2_ · 6 H2O 1, NaH_2_PO_4_ · H_2_O 1.25, CaCl_2_ · 2 H_2_O 2, Glucose 25, and NaHCO_3_ 25. Afterward, slices were kept at room temperature (between 21 °C and 23 °C) until recording. Cutting solution and ACSF were continuously infused with carbogen (95% O_2_, 5% CO_2_). All procedures were approved by the Norwegian Food Safety Authority (FOTS 6590, 7480, 19129, 30014), and experiments were performed in accordance with the Norwegian Animal Welfare Act.

***Patch-clamp recordings***: borosilicate glass pipettes were pulled using a PC-100 pipette puller (Narishige, Japan) to a resistance of 5 to 10 MΩ, and filled with an intracellular solution containing (in millimolar): K-gluconate 105, KCl 30, Na_2_-Phosphocreatine 10, Hepes 10, ATP-Mg 4, and GTP-Na 0.3. Neurons were visualized on a custom-built enhanced contrast upright microscope using a 40× long-working-distance immersion objective (Olympus, Japan). MSN identity was confirmed based on their electrophysiological properties, including hyperpolarized resting membrane potential, low spike-rate adaptation, and strong inward rectification. Paired data was obtained by recording an MSN at either room or physiological temperature, and maintaining the patch while temperature was adjusted.

MSNs were recorded in current-clamp mode on a MultiClamp 700B (Molecular Devices, United States), with a holding current applied to maintain a resting potential near − 75 mV. Additional voltage-clamp recordings were performed from a holding potential of −75mV. For a subset of voltage-clamp experiments, 1 µM tetrodotoxin was applied through the perfusate for at least 5 min prior to recording^24^. Data were recorded at 20 kHz using the WaveSurfer application^28^ built on Matlab 2020a (MathWorks, United States) and analyzed using custom scripts written for Matlab 2022b (MathWorks, United States).

Noise estimates were performed in Matlab by applying a 1-D digital filter with a rational transfer function of 0.5ms, or 10 samples, in length^29^. Raw signals were mean-adjusted to zero, filtered, and the filtered trace was subsequently subtracted from the raw signal. Standard deviations were calculated over 10 s of continuous noise.

Voltage-gated potassium currents were estimated by taking the weighted average of the leak current measured at −70mV, −65mV and − 60mV. A 150 Hz low-pass filter was applied to the leak current model prior to subtraction. Current maximums were determined over a 150ms window following the onset of each voltage step, and the first point where this current reached 1–1/e of said maximum was taken as the time constant of the voltage gated potassium current.

### Temperature measurements

Temperature measurements were performed using a calibrated TA612B multichannel thermometer (TASi group, United Kingdom), with one probe submerged in the recording chamber, and the other probe attached to the top of the heating block near the probe of the STC-1000 temperature controller. Temperature data was recorded at a rate of 0.1 Hz, except in a subset of experiments where data was recorded at 0.2 Hz (Supplementary Fig. 3).

### Component design and 3D printing

The spacer / mounting bracket was designed using AutoCAD 2022 (Autodesk, USA)^30^. Designs were exported as an STL file, and sliced using PrusaSlicer-2.5.2 (Prusa Research, Czech Republic)^31^. Printing was performed using Prusament PETG filament, on a Prusa i3 Mark 3 S (Prusa Research, Czech Republic) using a 5% infill setting for the spacer and 15% infill setting for the cover.

The aluminium heat exchanger was milled by a local workshop, to the design specified in supplementary Fig. 1. The four holes for mounting the heater to the breadboard were drilled slightly oversized to allow for dimensional inaccuracies; care must be taken when adopting this design for an imperial-unit breadboard, as the standard hole-sizing differs enough to preclude interoperability.

### Data analysis

All data are presented as mean ± SEM, unless otherwise specified. Statistical analysis was performed using SPSS 29.0.0.0 (IBM, United States)^32^and Origin 9.0 (OriginLab Corporation, United States)^33^. The number of replicates and P values are indicated in the text.

### Data availability

All source data are available from the corresponding author upon request. 3D print files are available on Zenodo at https://zenodo.org/records/11167885^18^.

## Electronic supplementary material

Below is the link to the electronic supplementary material.

Supplementary Material 1
