# Supplementary material for: A low-cost perfusion heating system for slice electrophysiology
Source: Sci Rep. 2024 Nov 18;14:28521. doi: 10.1038/s41598-024-79856-4 (PMC11574319; doi:10.1038/s41598-024-79856-4)
Supplement: Supplementary file 1 — Supplementary Material 1 [file 41598_2024_79856_MOESM1_ESM.docx]

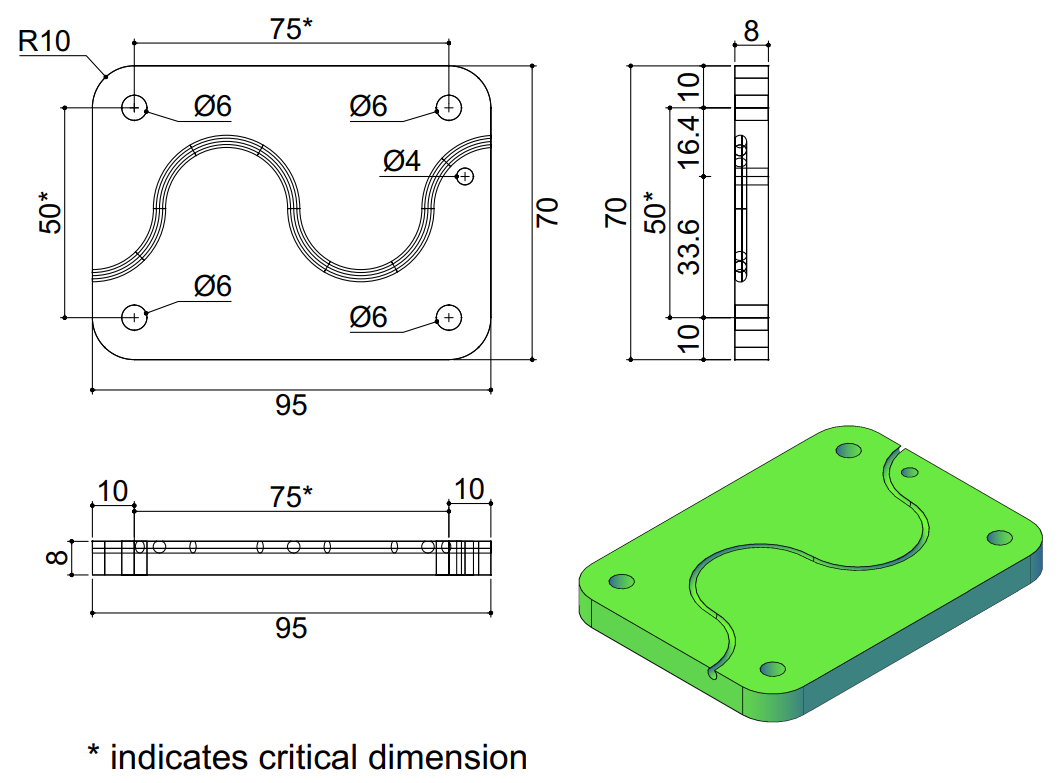


**Supplementary Figure 1.** Design schematics for the heat exchanger. All units in millimeters, critical dimensions are indicated with an asterisk (*).


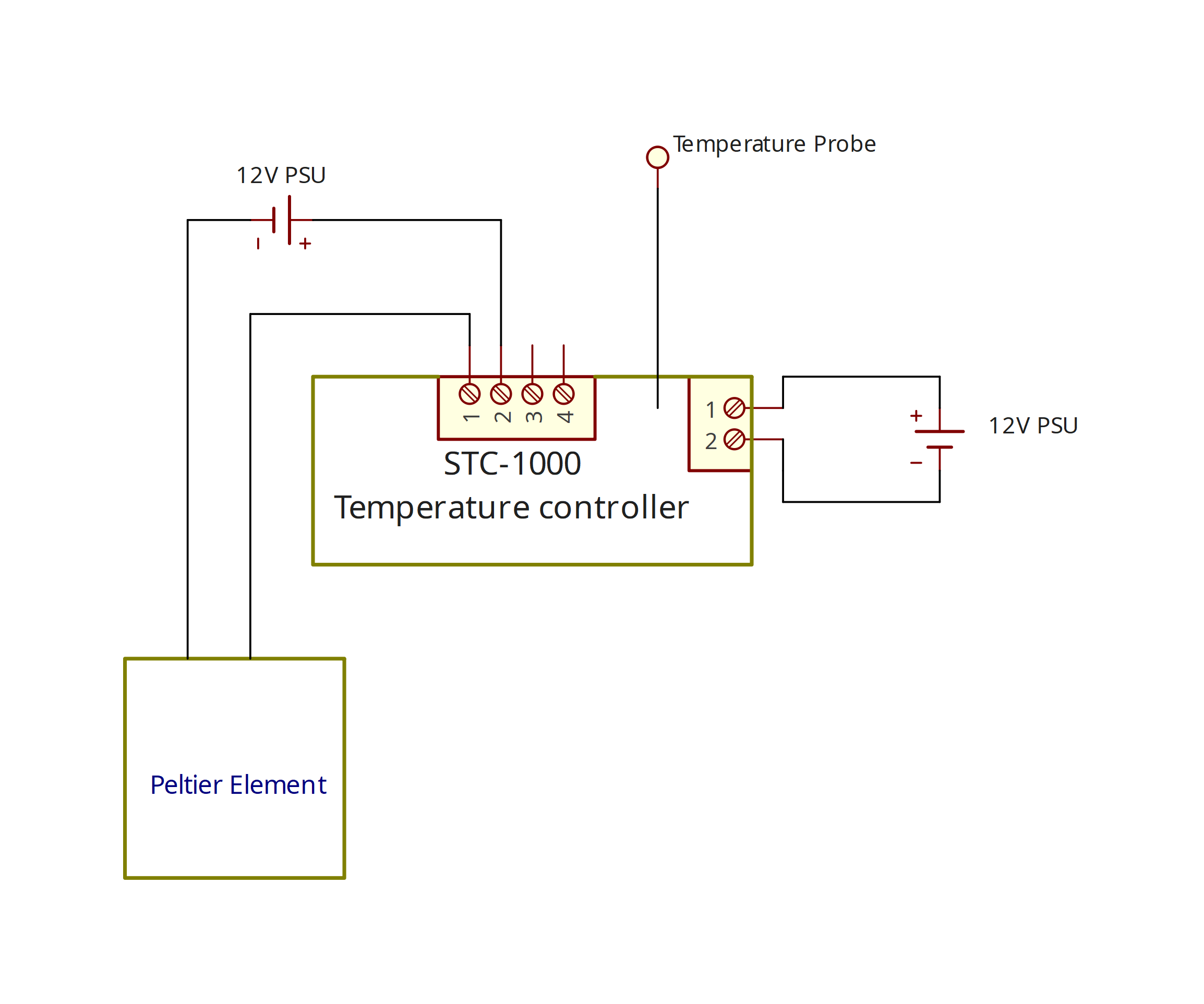


**Supplementary figure 2.** Wiring schematic for the peltier element, power supplies, and STC-1000 temperature controller. Care should be taken to connect the peltier element to the relay marked as “heating” or “cooling” on the temperature controller, as desired for the intended application.

**
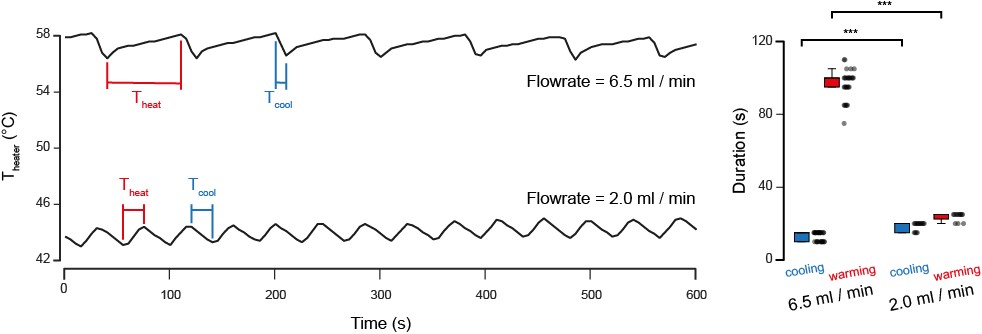
**

**Supplementary Figure 3: cycling time depends on flow rate. A.** heating and cooling cycles at perfusate flowrates of 6.5 ml / min and 2.0 ml / min were recorded at an acquisition rate of one sample per 5 seconds. **B.** Cooling cycles were significantly shorter at 12.9 seconds at a flowrate of 6.5 ml / min, compared to 18.7 seconds at the slower 2.0 ml / min flowrate (n = 28 / 15 cycles at high and low flow rates respectively, p = 1.02E-6, independent samples Mann-Whitney U test). Heating cycles were significantly longer at 96.4 seconds at a flowrate of 6.5 ml / min, compared to 23.7 seconds at 2.0 ml / min (n = 28 / 15 cycles at high and low flow rates respectively, p = 5.03E-8, independent samples Mann-Whitney U test).
